# Prediction of Glioma Grade and IDH Status Using ^18^F-FET PET/CT Dynamic and Multiparametric Texture Analysis

**DOI:** 10.3390/diagnostics13152604

**Published:** 2023-08-05

**Authors:** Rami Hajri, Marie Nicod-Lalonde, Andreas F. Hottinger, John O. Prior, Vincent Dunet

**Affiliations:** 1 Department of Diagnostic and Interventional Radiology, Lausanne University Hospital, Centre Hospitalier Universitaire Vaudois, 1011 Lausanne, Switzerland; rami.hajri@chuv.ch; 2 Department of Nuclear Medicine and Molecular Imaging, Lausanne University Hospital, Centre Hospitalier Universitaire Vaudois, 1011 Lausanne, Switzerland; marie.nicod-lalonde@chuv.ch (M.N.-L.); john.prior@chuv.ch (J.O.P.); 3 Department of Neurology, Lausanne University Hospital, Centre Hospitalier Universitaire Vaudois, 1011 Lausanne, Switzerland; andreas.hottinger@chuv.ch; 4 Lukas Lundin & Family Brain Tumor Research Center, Lausanne University Hospital, Centre Hospitalier Universitaire Vaudois, 1011 Lausanne, Switzerland

**Keywords:** gliomas, ^18^F-FET PET, radiomics, IDH mutational status, texture analysis

## Abstract

Mutations in isocitrate dehydrogenase (IDH) represent an independent predictor of better survival in patients with gliomas. We aimed to assess grade and IDH mutation status in patients with untreated gliomas, by evaluating the respective value of ^18^F-FET PET/CT via dynamic and texture analyses. A total of 73 patients (male: 48, median age: 47) who underwent an ^18^F-FET PET/CT for initial glioma evaluation were retrospectively included. IDH status was available in 61 patients (20 patients with WHO grade 2 gliomas, 41 with grade 3–4 gliomas). Time–activity curve type and 20 parameters obtained from static analysis using LIFEx© v6.30 software were recorded. Respective performance was assessed using receiver operating characteristic curve analysis and stepwise multivariate regression analysis adjusted for patients’ age and sex. The time–activity curve type and texture parameters derived from the static parameters showed satisfactory-to-good performance in predicting glioma grade and IDH status. Both time–activity curve type (stepwise OR: 101.6 (95% CI: 5.76–1791), *p* = 0.002) and NGLDM coarseness (stepwise OR: 2.08 × 10^43^ (95% CI: 2.76 × 10^12^–1.57 × 10^74^), *p* = 0.006) were independent predictors of glioma grade. No independent predictor of IDH status was found. Dynamic and texture analyses of ^18^F-FET PET/CT have limited predictive value for IDH status when adjusted for confounding factors. However, they both help predict glioma grade.

## 1. Introduction

Gliomas represent the most common form of primary brain tumor and their occurrence is increasing, especially among the elderly population, possibly due to the aging of the population, air pollution or ionizing radiation, among other factors [1,2,3,4]. According to the World Health Organization (WHO)’s 2021 classification of Tumors of the Central Nervous System (CNS) [5], gliomas are divided into low-grade (1–2) and high-grade tumors (3–4). Their histologic differentiation and grading are predictive of a patient’s outcome. Advances in oncogenetics have highlighted the role of the isocitrate dehydrogenase (IDH) genotype, notably in glioma oncogenesis and prognosis, as patients with an IDH mutation tend to have better outcomes [6]. IDH status also has implications for treatment, as the wide preponderance of IDH mutations has led to the development of new targeted agents that can inhibit these enzymes [7].

To date, no consensually approved method exists to determine IDH status in a noninvasive manner. Nevertheless, some studies using magnetic resonance imaging (MRI) radiomics [8] have shown high accuracy in predicting IDH status, with an area under the curve (AUC) > 0.9, using magnetic resonance (MR) spectroscopy with hydroxyglutarate, among other methods [9]. Conventional MRI represents the gold standard for the initial morphologic evaluation of a suspected brain tumor due to its high spatial resolution. MRI can assess the size, location and presence of complications such as mass effects or hemorrhages [10]. Advanced MRI has demonstrated its potential in distinguishing between low-and high-grade gliomas, notably due to the development of MR perfusion and diffusion-weighted imaging (DWI) [11].

In parallel, brain positron emission tomography (PET) using ^18^F-fluoro-ethyl-tyrosine (^18^F-FET), an artificial amino acid taken up by upregulated tumor cells due to their increased expression of LAT1 and LAT2 amino acid transporters [12,13], has played an increasing role in the initial evaluation of gliomas. It provides information on tumor metabolism by identifying the most hypermetabolic zones to help with biopsy targeting, for example, and has also demonstrated excellent diagnostic and prognostic value [14].

The combination of MRI techniques [15] or of PET and MRI techniques yields different information [16,17] and has shown additional value compared to each technique used alone [10], notably due to the development of hybrid PET/MRI systems. Beyond morphological and ^18^F-FET uptake evaluation, several advanced image post-processing techniques have demonstrated their usefulness, particularly the evaluation of the dynamic TAC of ^18^F-FET PET; the analysis of apparent diffusion coefficient (ADC) histograms from DWI; the textural analysis of dynamic susceptibility contrast (DSC) and static ^18^F-FET images, as they provide important information for the diagnosis, grading and prognosis of gliomas [10,18,19,20]; and the analysis of the prediction of the IDH genotype [21,22,23].

More specifically, texture analysis in the context of radiomics is an emerging field with fast development potential. It consists in extracting mathematically defined features from medical images, and obtaining quantitative information that allows for the identification of parameters regarding tumor heterogeneity and improves diagnostic and prognostic accuracy [22].

We evaluated the respective and combined value of ^18^F-FET PET/CT dynamic and texture analyses to predict glioma grade and IDH genotype.

## 2. Materials and Methods

### 2.1. Study Population

In this retrospective monocentric study, 73 patients with suspected primary brain tumors based on conventional MRI who underwent supplemental ^18^F-FET PET/CT for the initial evaluation of gliomas between 2009 and 2019 were included. All the examinations were performed before treatment.

**Figure 1:**
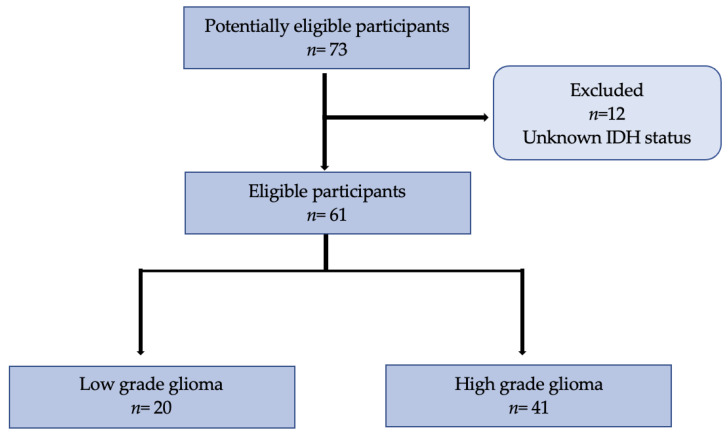
Flowchart of 73 participants included in the study with 12 excluded initially for unavailable IDH status. In total, 61 participants were eligible with an available tumor grade and IDH status, from which 20 were classified as low-grade and 41 as high-grade.

All patients underwent histopathological confirmation via either surgical stereotactic biopsy (*n* = 43) or tumor neurosurgical resection (*n* = 30). Tumors were then histologically classified according to the 2021 WHO classification of Tumors of the Central Nervous System [5]. The histological parameters recorded were the tumor type, tumor grade and IDH status. Patients whose IDH status was unknown were excluded from the statistical analysis (Figure 1). The study was conducted according to the Standards for Reporting Diagnostic Accuracy Studies (STARD) criteria [24]. All collected imaging data were anonymized to comply with national ethical guidelines and according to the Swiss Federal Act on Research involving Human Beings from 2011 (HRA, Art. 3). The study protocol (196/08 and 2018/01513) was approved by the State of Vaud Ethics Committee and Federal Regulatory Agencies. Each participant gave written informed consent before inclusion.

### 2.2. ^18^F-FET PET Acquisitions

^18^F-FET PET acquisitions were performed using a PET/CT scanner (Discovery LS or 690, GE Healthcare, Milwaukee, MI, USA). All patients fasted, avoiding any intake for at least 4 h prior to intravenous ^18^F-FET injection, as recommended by European Association of Nuclear Medicine (EANM) guidelines [25].

^18^F-FET PET images were acquired using a dynamic protocol over 50 to 60 min (12 frames of 5 min; 4.2 mm section thickness; 24 cm field-of-view; matrix size of 256 × 256) after intravenous injection of 185–250 MBq of ^18^F-FET. Raw data were corrected for attenuation by soft tissue and skull bone using an unenhanced cerebral CT (120 kV, 10 mAs) and normalized to the injected dose and body mass by calculating the standardized uptake value (SUV). Other raw data corrections were performed, such as time-of-flight, scatter correction, random correction and dead time correction. No head motion correction was needed.

### 2.3. ^18^F-FET PET Analysis

Before being analyzed, all data were anonymized. Analyses were performed independently by two readers (RH, VD) blinded to the histological results. One reader (RH) with 4 years’ experience in neuroimaging analyzed the ^18^F-FET PET static data and one reader (VD) with 17 years’ experience in neuroimaging analyzed the ^18^F-FET PET dynamic data.

Static ^18^F-FET PET data were analyzed using the free LIFEx© [26] v6.30 software, which enables the extraction of several parameters obtained from the static PET data (conventional, texture, shape and histogram-derived). For each patient, the extraction of texture features was performed after contouring the tumoral volume-of-interest (VOI) using a semi-automated method based on a tumor-to-background ratio (TBR) threshold value. For this purpose, a spherical VOI was placed in normal-looking contralateral brain tissue as a reference to determine the SUVmean background. Of the 61 patients analyzed, 45 patients had their static data obtained over a 40–50 min window, and 16 patients over a 40–60 min window. The static ^18^F-FET at 40–50 or 40–60 min post-injection was then normalized to obtain a TBR map, and the tumor VOI was automatically delineated using a TBR ≥ 1.6 [27]. TBRmean and TBRmax were thus calculated by dividing the mean SUVmean or SUVmax of the tumor VOI by the mean SUV of contralateral normal brain tissue, respectively.

LIFEx© software computed 15 textural parameters that differ from each other in the way they are mathematically calculated [28]. The grey-level co-occurrence matrix (GLCM) takes into account the arrangements of pairs of voxels to calculate textural indices. The GLCM is calculated from 13 different directions in 3D with a δ-voxel distance (k − → dk) relationship between neighboring voxels. The index value is the average of the index over the 13 directions in space (X, Y, Z). Six textural indices are computed from this matrix (homogeneity, energy, contrast, correlation, entropy and dissimilarity).

The neighborhood grey-level difference matrix (NGLDM) corresponds to the differences in grey levels between one voxel and its 26 neighbors in 3 dimensions (8 in 2D). Three texture indices can be computed from this matrix (coarseness, contrast, busyness). Indices from shape were also extracted as four parameters, including sphericity, surface, compacity and volume. Finally, two parameters were extracted from the first-order features from histogram data and consisted of skewness and kurtosis.

**Figure 2:**
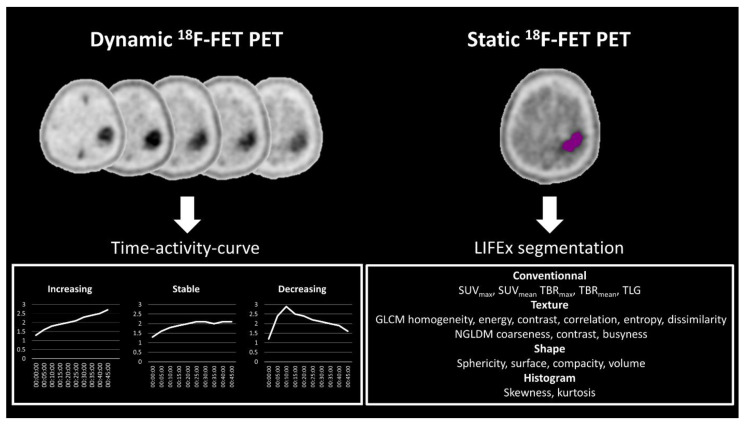
Post-processing pipeline included analysis of the time–activity curves derived from the dynamic ^18^F-FET PET data on the left with three types: increasing, stable and decreasing. On the right, the texture parameters are shown that were obtained from the static analysis using the LIFEx^©^ software. SUV, standardized uptake value; TBR, tumor-to-background ratio; TLG, total lesion glycolysis; GLCM, grey-level co-occurrence matrix; NGLDM, neighboring gray-level dependence matrix.

Dynamic ^18^F-FET PET analysis was performed using Syngo.via© version VB50 software (Siemens Healthineers, Erlangen, Germany). From the dynamic data, the time–activity curve (TAC) type (i.e., increasing, stable or decreasing) was recorded (Figure 2).

### 2.4. Statistical Analysis

The statistical analysis was performed using Stata 16.0 software (Stata, College Station, TX, USA). Continuous variables are reported as median (interquartile range (IQR)) and categorical variables as number (percentage). We divided patients according to their histopathological grade (low-grade versus high-grade) and IDH status (wildtype versus mutated). The diagnostic performance of all metrics was assessed using receiver operating characteristic (ROC) curve analysis with computation of the area under the curve (AUC) with a 95% confidence interval (95% CI). The diagnostic performance of all metrics was also compared with the Chi-squared test of equality of the AUC.

Stepwise multivariate regression analysis was additionally performed to identify independent predictors that could be combined for tumor grade and IDH status estimation, while adjusting for patients’ age and sex, which are confounding factors [29,30]. A *p*-value < 0.05 was considered statistically significant. The significance level was corrected for multiple testing when necessary.

## 3. Results

### 3.1. Study Population

Overall, 73 patients (female 25; male 48) were enrolled in this study with a median age of 47 years (36–59). A total of 12 patients were excluded from the analysis as their IDH statuses were not available. In total, 61 patients (female: 20, male: 41, median age: 47 years (35–57)) could be fully classified according to the 2021 WHO classification. A total of 20 patients (33%) had a WHO grade 2 gliomas and 41 patients (67%) had a grade 3–4 gliomas. Of the 20 patients with a grade 2 glioma, 9 (15%) had an astrocytoma and 11 (18%) an oligodendroglioma. Of the 41 patients with high-grade gliomas, 3 (5%) had an astrocytoma, 10 (16%) had an oligodendroglioma, and 28 (46%) had a glioblastoma. Regarding IDH status, 28 patients had wildtype (wt) IDH (46%), all corresponding to glioblastomas, and 33 patients had mutant IDH (54%), as displayed in Table 1.

### 3.2. Dynamic ^18^F-FET PET Analysis

Of the 61 patients, 30 (49%) patients had a decreasing time–activity curve (TAC) and 31 (51%) had a stable or increasing time–activity curve. TAC type showed good performance in distinguishing between low-grade and high-grade gliomas (AUC = 0.80 [0.69–0.91]) and moderate performance in IDH status identification (AUC = 0.67 [0.55–0.79]). In addition, it was shown to be an independent predictor of glioma grade (stepwise OR: 101.6 (95% CI: 5.76–1791), *p* = 0.002) but not of IDH status. There was no significant difference in TAC type between tumor types of the same grade, notably in low-grade tumors (*p* = 0.42) and high-grade tumors (*p* = 0.20).

### 3.3. Static ^18^F-FET PET Texture Analysis

Static ^18^F-FET PET texture analysis was successfully performed in all 61 patients with an available IDH status. The diagnostic performance of the 20 parameters used for grading and IDH status assessment, which were derived from the analysis, including conventional metrics, texture features, indices from shape and first-order features from histogram, is presented in Table 2.

For glioma grading, NGLDM coarseness, NGLDM contrast and sphericity had good-to-satisfactory performance. The NGLDM coarseness was the only factor that independently predicted glioma grade (stepwise OR: 2.08 × 10^43^ (95% CI: 2.76 × 10^12^–1.57 × 10^74^), *p* = 0.006). Despite their moderate performance in identifying IDH status, as shown in Table 3, the other parameters were not independent predictors of IDH status in the stepwise regression analysis.

There was no difference in parameter performance for identifying either glioma grade or IDH status between the two time windows (i.e., 40–50 versus 40–60 min, all *p*-values > 0.093).

### 3.4. Performance Comparison of Dynamic ^18^F-FET PET and Static Texture Analysis

Regarding the performance of both techniques for glioma grading, the ROC comparison did not find a significant difference between TAC, NGLDM contrast, NGLDM coarseness and sphericity (*p* > 0.05), but TAC had better performance than all the other parameters (*p* < 0.046).

**Figure 3:**
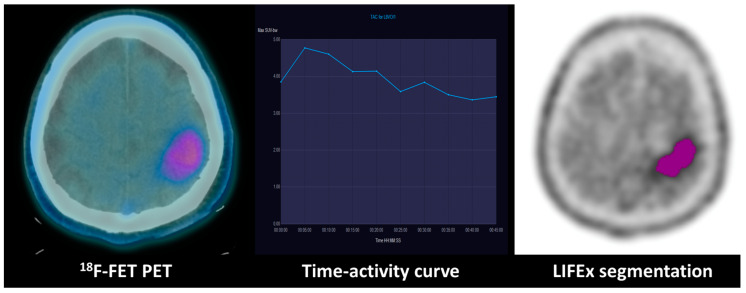
Combination of dynamic curve and segmentation study of patient with WHO grade III anaplastic astrocytoma: ^18^F-FET PET uptake in the left fronto-parietal lobe of a WHO grade III anaplastic astrocytoma with an IDH mutation. The time–activity curve shows a decreasing pattern, and the GLCM (grey-level co-occurrence matrix) correlation extracted from the tumoral VOI texture analysis evidenced on the right is 0.67.

**Figure 4:**
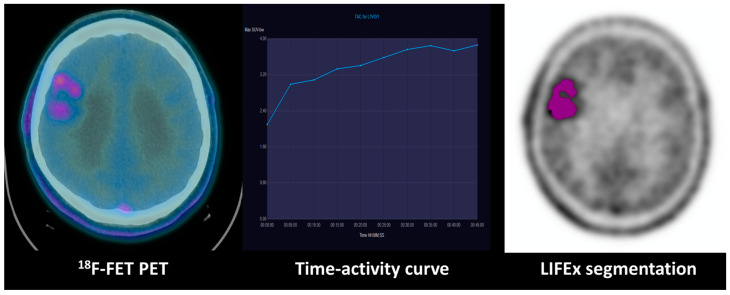
Combination of dynamic curve and segmentation study of a patient with WHO grade II astrocytoma: ^18^F-FET PET uptake in the right frontal lobe of a WHO grade II astrocytoma with an IDH mutation. The time–activity curve shows an increasing pattern, and the GLCM (grey-level co-occurrence matrix) correlation extracted from the tumoral VOI texture analysis evidenced on the right is 0.73.

Regarding the performance of both techniques for identifying IDH status, the ROC comparison did not find significant differences between TAC and several texture parameters, especially NGLDM contrast, coarseness and sphericity (*p* > 0.90). Exemplary cases of low-grade and high-grade gliomas with TAC type and relevant texture parameters are displayed in Figure 3 and Figure 4.

## 4. Discussion

In this study, we assessed the respective value of ^18^F-FET PET/CT dynamic and static texture analyses regarding the prediction of 2021 WHO glioma grade and IDH status. Our results show that TAC type and several static parameters derived from texture analysis have good-to-moderate performance in identifying glioma grade and IDH status. In addition, both TAC type and NGLDM coarseness are independent predictors of glioma grade. However, neither TAC type nor texture parameters are independent predictors of IDH status. Hence, the combination of ^18^F-FET PET/CT dynamic and texture analyses may help to predict glioma grade but has little value for predicting IDH status.

The fifth edition of the WHO Classification of Tumors of the CNS published in 2021 introduced major changes that advanced the role of molecular diagnostics in tumor classification [5]. The previous CNS tumor classifications were based exclusively on histological features determined via immunohistochemistry. The 2016 WHO classification introduced molecular markers, including the mutational status of IDH, for the first time as a component of glioma classification. In the current WHO classification determined in 2021, the role of molecular biomarkers has increased in importance and provides powerful clinicopathologic information and utility for more accurate classification, prognosis and management. For example, IDH-wildtype gliomas and glioneuronal and neuronal tumors now extend from CNS grade 2 or 3 to CNS grade 4, even in cases that previously would appear histologically as a lower grade. This highlights the importance of determining IDH status to classify patients according to the fifth edition of the WHO CNS classification of gliomas and to provide the most well-adapted treatment as a consequence. The current guidelines recommend the determination of IDH status for grading and to predict outcomes and responses to therapy [5]. However, to date, no consensually accepted method allows for the identification of IDH status in a non-invasive manner.

The applications of ^18^F-FET PET in neuro-oncology, especially in gliomas, are increasing, as its use has been recommended by the Response Assessment in Neuro-Oncology (RANO) Working Group and the European Association of Neuro-Oncology (EANO) as a complementary tool to contrast-enhanced MRI [31,32]. ^18^F-FET PET is highly relevant to the initial diagnosis of primary brain tumors and gliomas (14) and outperforms ^18^F-FDG PET in this setting [33]. Beyond tumor tracer uptake, previous studies have demonstrated the diagnostic performance of the time–activity curve derived from dynamic ^18^F-FET PET for glioma grading [21]. Dunet et al. [10] also demonstrated the added value of dynamic ^18^F-FET PET for the initial grading of untreated gliomas using the ^18^F-FET time–activity curve pattern, alone or in combination with the apparent diffusion coefficient histogram derived from diffusion-weighted MRI.

In the present study, we confirm the independent predictive value of the ^18^F-FET time-activity curve for identifying high-grade gliomas. However, we found that the time–activity curve type has only moderate performance and is not an independent predictor of IDH mutation when adjusting for patients’ age and sex. The parameters derived from dynamic ^18^F-FET PET curves were described to be useful for IDH status determination [22]. However, the authors reported no correction for patients’ age and sex, with their influence on the results remaining unclear in their study. Suchorska et al. [34] showed that ^18^F-FET-derived dynamic analysis defines prognostically distinct subgroups of IDH mutant −1p/19q non-codeleted gliomas. Moreover, Li et al. [35] showed that radiomics based on time-to-peak images extracted from dynamic ^18^F-FET PET could predict the TERTp-mutation status of IDH-wildtype diffuse astrocytic high-grade gliomas (now called glioblastomas according to the 2021 WHO CNS classification) [2] with high accuracy preoperatively. In addition, Vomacka et al. [36] suggested that voxel-wise analysis of dynamic ^18^F-FET PET could help identify aggressive tumor areas and guide individual management. This was confirmed by Blanc-Durand et al. [21] who further demonstrated that tumor heterogeneity evaluated via a clustering approach to the time–activity curve is a valuable method in differentiating IDH mutation status and for survival stratification. However, whether static ^18^F-FET PET texture analysis could replace dynamic ^18^F-FET PET to reduce scan time remains unknown. In patients with IDH mutation, another element that could be evaluated through further studies is the value of radiomics analysis for estimating the CDKN-2A/B deletion, which is known as a supplementary prognostic factor [37].

In recent decades, lesion heterogeneity, which is thought to be the hallmark of aggressive tumors, has been increasingly studied, notably using image textural feature analysis [38]. Thanks to advances in image acquisition standardization [39] and efforts in reproducible radiomics analyses made by the Image Biomarker Standardization Initiative (IBSI) [40], ^18^F-FET textural feature analysis has been proven highly repeatable, regardless of IDH genotype [41]. In the present study, we used the free LIFEx© software, which ensures both standardized and reproducible data analysis. We found that several textural features derived from ^18^F-FET static images had good-to-substantial performance either for glioma grading or for IDH status identification. Notably, NGLDM coarseness, which is the level of the spatial rate of change in intensity, was an independent predictor of glioma grade. High-grade gliomas indeed had higher NGLGM coarseness values, which indicates higher heterogeneity. This is in line with Hua et al. [42], who demonstrated higher coefficient of variation and heterogeneity indices in patients with IDH-wildtype gliomas evaluated via 20–40 min static ^18^F-FET PET. Pyka et al. [18] also reported that several textural features, notably coarseness, might help to distinguish between grade 3 and grade 4 gliomas, as well as predicting progression-free survival and overall survival. However, these authors did not report IDH status and used non-standard contouring and calculating methods, which precludes any direct comparison. Compared to the TAC analysis, the performances of NGLGM coarseness and contrast, and sphericity, were similar for the determination of IDH status in our study. None were independent predictors. Lohmann et al. [22] evaluated the performance of ^18^F-FET PET/MRI textural parameters recorded using the LIFEx© software in a population of 28 patients with untreated gliomas. The authors demonstrated that, among various combination of parameters, combining decreasing TAC and NGLDM contrast or decreasing TAC and sphericity improves the non-invasive prediction of the IDH genotype. Again, potential confounding factors such as age or sex were not evaluated, making any comparison difficult. Overall, this suggests that TAC analysis is not outperformed by textural feature analysis, but its respective value for IDH status determination appears to be small. Larger studies considering confounding factors are needed to refine the place of ^18^F-FET PET texture analysis for the initial evaluation of patients with untreated gliomas.

Beyond ^18^F-FET PET/CT, the use of combined PET-MR imaging is expanding in the field of brain tumor imaging as they both provide valuable information regarding biological characteristics. The increasing availability of hybrid PET/MRI machines that allow for the concurrent acquisition of both modalities is possible thanks to the many studies that have addressed the benefit of the combination of both techniques [43]. For instance, Verger et al. [17] investigated the usefulness of ^18^F-FET PET and dynamic susceptibility contrast-enhanced perfusion weighted imaging (DSC-PWI) at 3 Tesla for the grading of gliomas and evidenced that its diagnostic accuracy for glioma grading was comparable for both ^18^F-FET PET and relative cerebral blood volume (rCBV), with an AUC of about 0.80. Song et al. [44] also showed that both ^18^F-FET PET and DSC-PWI could be non-invasive predictors of glioma grades and IDH status. Combining dynamic and textural ^18^F-FET PET analyses with multiparametric MRI might still improve the characterization of untreated gliomas.

The main strength of this study is the standardization of the methodology through the use of LIFEx© software in the acquisition of the texture analysis parameters, which has been shown to be highly robust [26]. Also, our study highlights that TAC analysis and texture analysis are valuable for glioma grading but have little value for IDH prediction, when considering potential confounding factors such as age and sex, which has not been addressed in many other studies published in the field [22,45,46]. One limitation of our study is its monocentric retrospective design with a selected population of patients with gliomas, not including benign lesions and non-glial tumors. In addition, we did not have the IDH status of 12 of the 73 enrolled patients. Another limitation is the use of different PET-CT machines. However, a phantom was used to ensure the stability of SUV measures, hence the reproducibility of the measurements [47]. Moreover, the delineation of the tumor VOI was realized using the static ^18^F-FET PET images from the 40–50 or 40–60 min acquisition according to the protocol, which prevailed at the time of the study, and not according to the recent EANM/EANO/RANO/Society of Nuclear Medicine and Molecular Imaging (SNMMI) practice guidelines for the imaging of gliomas using PET with radiolabeled amino acids and ^18^F-FDG [48]. Nevertheless, there was no statistical difference in parameter performance between the 40–50- and 40–60-min windows in determining either glioma grade or IDH status (all *p*-values > 0.093). The diagnostic impact of performing texture analysis on 40–60 min versus 20–40 min images could be evaluated but was not possible here, and is of interest for future studies.

## 5. Conclusions

Our results indicate that dynamic and texture analyses of ^18^F-FET PET/CT have only limited predictive value for IDH status when adjusted for confounding factors. However, they both help predict glioma grade.

## Figures and Tables

**Table 1:** Histological characteristics of tumors according to 2021 WHO classification.

2021 WHO Classification	Grade 2	Grade 3–4	Total
Astrocytoma	9	3	12
Oligodendroglioma	11	10	21
Glioblastoma	0	28	28
	**IDH wt**	**IDH mutant**	
Astrocytoma	0	12	12
Oligodendroglioma	0	21	21
Glioblastoma	28	0	28

**Table 2:** Performance in glioma grading based on 2021 WHO classification.

Parameters	AUC	95% CI	*p*-Value *
*Dynamic*			
TAC	0.80	0.69–0.91	NA
*Conventional static*			
SUVmax	0.58	0.42–0.74	0.010
SUVmean	0.54	0.38–0.70	0.0052
TBRmax	0.56	0.39–0.73	0.0045
TBRmean	0.56	0.40–0.73	0.0071
TLG	0.57	0.40–0.74	0.0078
*Texture features*			
GLCM Homogeneity	0.41	0.24–0.58	0.0003
GLCM Energy	0.44	0.26–0.62	0.0024
GLCM Contrast	0.60	0.43–0.76	0.037
GLCM Correlation	0.39	0.21–0.58	<0.0001
GLCM Entropy	0.55	0.37–0.73	0.012
GLCM Dissimilarity	0.60	0.43–0.77	0.046
NGLDM Coarseness	0.73	0.57–0.89	0.54
NGLDM Contrast	0.66	0.49–0.83	0.22
NGLDM Busyness	0.40	0.22–0.58	0.0002
*Indices from shape*			
Sphericity	0.63	0.46–0.80	0.11
Surface	0.41	0.22–0.60	0.0001
Compacity	0.51	0.30–0.71	0.0034
Volume	0.55	0.37–0.72	0.0044
*First-order features from Histogram*			
Skewness	0.40	0.24–0.55	0.0001
Kurtosis	0.39	0.23–0.54	0.0001

AUC, area under the curve from receiver operating characteristics; 95% CI, 95% confidence interval; TAC, time–activity curve; SUV, standardized uptake value; TBR, tumor-to-background ratio; TLG, total lesion glycolysis; GLCM, grey-level co-occurrence matrix; NGLDM, neighboring gray-level dependence matrix. Test for grade 2 (*n* = 20) versus grade 3–4 (*n* = 41). * *p*-value in comparison to TAC type. ROC comparison did not find significant differences between TAC, NGLDM contrast, NGLDM coarseness and sphericity (*p* > 0.05) but TAC had better performance than all the others parameters (*p* < 0.046).

**Table 3:** Performance in identifying IDH status.

Parameters	AUC	95% CI	*p*-Value *
*Dynamic*			
TAC	0.67	0.55–0.79	NA
*Conventional static*			
SUVmax	0.56	0.41–0.71	0.18
SUVmean	0.56	0.42–0.71	0.27
TBRmax	0.47	0.32–0.62	0.0048
TBRmean	0.45	0.30–0.60	0.0028
TLG	0.47	0.32–0.62	0.0070
*Texture features*			
GLCM Homogeneity	0.45	0.30–0.61	0.066
GLCM Energy	0.50	0.34–0.66	0.18
GLCM Contrast	0.53	0.37–0.70	0.21
GLCM Correlation	0.43	0.26–0.59	0.014
GLCM Entropy	0.51	0.35–0.67	0.11
GLCM Dissimilarity	0.54	0.38–0.70	0.23
NGLDM Coarseness	0.62	0.47–0.78	0.91
NGLDM Contrast	0.64	0.49–0.80	0.90
NGLDM Busyness	0.47	0.31–0.63	0.079
*Indices from shape*			
Sphericity	0.65	0.49–0.80	0.92
Surface	0.34	0.19–0.50	0.0003
Compacity	0.44	0.28–0.60	0.011
Volume	0.45	0.30–0.59	0.0022
*First-order features from Histogram*			
Skewness	0.48	0.33–0.63	0.080
Kurtosis	0.48	0.33–0.62	0.080

AUC, area under the curve from receiver operating characteristics; 95% CI, 95% confidence interval; TAC, time–activity curve; SUV, standardized uptake value; TBR, tumor-to-background ratio; TLG, total lesion glycolysis; GLCM, grey-level co-occurrence matrix; NGLDM, neighboring gray-level dependence matrix. Test performed on all gliomas (*n* = 61) and each group: glioblastoma IDH wt (*n* = 28), astrocytoma and oligodendroglioma IDH mutant (*n* = 33). * *p*-value in comparison to TAC type. ROC comparison did not find significant difference between TAC and several texture parameters, especially NGLDM contrast, coarseness and sphericity (*p* > 0.90).

## Data Availability

For detailed research data, please contact the corresponding author.
